# Network Efficiency and Posterior Alpha Patterns Are Markers of Recovery from General Anesthesia: A High-Density Electroencephalography Study in Healthy Volunteers

**DOI:** 10.3389/fnhum.2017.00328

**Published:** 2017-06-28

**Authors:** Stefanie Blain-Moraes, Vijay Tarnal, Giancarlo Vanini, Tarik Bel-Behar, Ellen Janke, Paul Picton, Goodarz Golmirzaie, Ben J. A. Palanca, Michael S. Avidan, Max B. Kelz, George A. Mashour

**Affiliations:** ^1^School of Physical and Occupational Therapy, Faculty of Medicine, McGill University; ^2^Center for Consciousness Science, University of Michigan Medical SchoolAnn Arbor, MI, United States; ^3^Department of Anesthesiology, University of Michigan Medical SchoolAnn Arbor, MI, United States; ^4^Department of Anesthesiology, Washington University School of MedicineSt. Louis, MO, United States; ^5^Department of Anesthesiology, University of PennsylvaniaPhiladelphia, PA, United States; ^6^Neuroscience Graduate Program, University of Michigan Medical SchoolAnn Arbor, MI, United States

**Keywords:** consciousness, cognition, general anesthesia, electroencephalography, alpha rhythm, graph theory

## Abstract

Recent studies have investigated local oscillations, long-range connectivity, and global network patterns to identify neural changes associated with anesthetic-induced unconsciousness. These studies typically employ anesthetic protocols that either just cross the threshold of unconsciousness, or induce deep unconsciousness for a brief period of time—neither of which models general anesthesia for major surgery. To study neural patterns of unconsciousness and recovery in a clinically-relevant context, we used a realistic anesthetic regimen to induce and maintain unconsciousness in eight healthy participants for 3 h. High-density electroencephalogram (EEG) was acquired throughout and for another 3 h after emergence. Seven epochs of 5-min eyes-closed resting states were extracted from the data at baseline as well as 30, 60, 90, 120, 150, and 180-min post-emergence. Additionally, 5-min epochs were extracted during induction, unconsciousness, and immediately prior to recovery of consciousness, for a total of 10 analysis epochs. The EEG data in each epoch were analyzed using source-localized spectral analysis, phase-lag index, and graph theoretical techniques. Posterior alpha power was significantly depressed during unconsciousness, and gradually approached baseline levels over the 3 h recovery period. Phase-lag index did not distinguish between states of consciousness or stages of recovery. Network efficiency was significantly depressed and network clustering coefficient was significantly increased during unconsciousness; these graph theoretical measures returned to baseline during the 3 h recovery period. Posterior alpha power may be a potential biomarker for normal recovery of functional brain networks after general anesthesia.

## Introduction

In recent years, the electroencephalographic study of anesthetic state transitions has focused on (1) coherent oscillations (Akeju et al., 2014), (2) functional connectivity (Lee U. et al., 2013), and (3) network analysis (Lee H. et al., 2013; Chennu et al., 2016). A number of findings have emerged that appear to distinguish states of consciousness from anesthetic-induced unconsciousness. For example, the shift of alpha power (Berger, 1930) from the occipital to frontal cortex—a process known as anteriorization and first posited to be a marker of general anesthesia based on nonhuman primate studies in the 1970s (Tinker et al., 1977)—is associated with propofol-induced unconsciousness and surgical levels of sevoflurane anesthesia (John et al., 2001; Purdon et al., 2013; Akeju et al., 2014). Functional disconnection of anterior and posterior cortical regions—first posited to be an invariant marker of general anesthesia in 2001 (John et al., 2001)—has been found to correlate with propofol-, sevoflurane-, and ketamine-induced unresponsiveness by both electroencephalography (EEG) and functional magnetic resonance imaging (fMRI) (Boveroux et al., 2010; Jordan et al., 2013; Lee U. et al., 2013; Palanca et al., 2015; Bonhomme et al., 2016; Ranft et al., 2016). Finally, graph theoretical network properties—first shown to be modulated by anesthetics in 2010 (Lee et al., 2010)—have been shown by both EEG and fMRI investigations to be disrupted during unconsciousness induced by propofol, dexmedetomidine, and halogenated ethers (Lee et al., 2011; Schröter et al., 2012; Moon et al., 2015; Hashmi et al., 2017).

Although, considerable progress has been made in identifying changes in key local oscillations, long-range connectivity, and global network patterns, many of the recent high-resolution neuroimaging studies of anesthetic-induced unconsciousness have been conducted in healthy volunteers with protocols that either (1) just cross the threshold of unconsciousness, or (2) induce more profound unresponsiveness but for a brief period of time. These conditions do not model general anesthesia for major surgery, which has prolonged periods of anesthetic exposure at concentrations far higher than those required to induce unconsciousness. One of the critical limitations of either “long but light” or “deep but brief” anesthetic protocols in healthy volunteers is that the recovery process is far more rapid than might be observed in the perioperative domain. Furthermore, neither the topography of oscillations nor the topology of networks has typically been studied beyond the initial return of consciousness. Collectively, these limitations have resulted in incomplete knowledge of the neural correlates of the recovering brain after the major perturbation of general anesthesia.

One advantage to the study of healthy volunteers is the absence of surgical stress, inflammatory burden, or polypharmacy, which confound neuroscientific insight into the recovery of consciousness and cognition after general anesthesia. Thus, what is needed is a protocol with surgically-relevant anesthetic regimens without surgical intervention, followed by neural data acquisition that extends far beyond the initial return of consciousness. In this study, we report the implementation of just such a paradigm, in which healthy human participants underwent induction of anesthesia with propofol, 3 h of age-adjusted 1.3 minimum alveolar concentration (MAC) of isoflurane anesthesia, followed by recovery and continued high-density EEG acquisition for another 3 h after emergence. These data were analyzed with source-localized spectral analysis, functional connectivity, and graph-theoretical approaches, testing the hypothesis that the full recovery of local and global network function is prolonged after anesthetic exposure simulating surgical conditions.

## Materials and methods

This study was conducted at the University of Michigan Medical School and approved by the Institutional Board Review (HUM0071578); written informed consent was obtained from all participants.

### Study population

We analyzed data from eight healthy volunteers (5 males, 23–29 year of age) with 128-channel EEG as a subset of the Reconstructing Consciousness and Cognition (ReCCognition) study (NCT01911195). The full protocol for this investigation has been published (Maier et al., 2017). Participants were American Society of Anesthesiologists class 1 physical status, body mass index <30, with Mallampati 1 or 2 airway classifications, and no other factors predictive of difficult airway. We excluded subjects who were pregnant, had a history of obstructive sleep apnea, reactive airway disease, neuropsychiatric disorders, history or current use of psychotropic medications, gastroesophageal reflux, cardiac conduction abnormalities, asthma, epilepsy, history of problems with anesthesia, family history of problems with anesthesia, and any neurologic or psychiatric history. Pregnancy and illicit drug use were ruled out through both urine and blood analyses.

### Anesthetic protocol

Induction and maintenance of general anesthesia with, respectively, propofol and a halogenated ether was chosen because of its relevance to routine clinical care. Isoflurane was chosen as the halogenated ether because the associated recovery would be longer than sevoflurane or desflurane, allowing more opportunity for the observation of differential recovery of cognitive and network function. Participants were assessed throughout the experiment by at least two anesthesiologists and standard monitors (i.e., oxygen saturation, noninvasive blood pressure, electrocardiogram, end-tidal carbon dioxide; nasal temperature probe). Participants were pre-oxygenated with 100% O_2_ by face mask and received intravenous propofol at increasing infusion rates over three consecutive 5-min blocks (block 1: 100 μg/kg/min, block 2:200 μg/kg/min, block 3:300 μg/kg/min). To assess loss of consciousness, a pre-recorded auditory instruction (i.e., “Squeeze your left/right hand twice,” with left or right randomized) was triggered every 30 s; the onset of anesthetic-induced unconsciousness was defined as the absence of response to two consecutive commands delivered 30 s apart. Isoflurane anesthesia was then administered with air and 40% oxygen at 1.3 age-adjusted minimum alveolar concentration (i.e., the ED_95_; Nickalls and Mapleson, 2003) via mask inhalation. A laryngeal mask airway was inserted and positive pressure ventilation was used to maintain tidal volumes at greater than 5 mL/kg and normocapnia (end-tidal carbon dioxide targeted to 35–45 mmHg). Surface warming blankets were applied to maintain normothermia, and phenylephrine was titrated as needed to maintain mean arterial pressure within 20% of pre-anesthetic values. Ondansetron 4 mg was administered 30-min prior to discontinuation of isoflurane. After 3 h of exposure to isoflurane, the anesthetic was discontinued, and participant responsiveness was assessed every 30 s using the same verbal command until the participant regained consciousness; recovery of consciousness (ROC) was defined as two consecutive responses to the command. The laryngeal mask airway was removed at or before ROC, as determined by the clinical anesthesia team.

### Electroencephalography data acquisition and preprocessing

The EEG was acquired using a 128-channel system from Electrical Geodesics, Inc. (Eugene, OR) with all channels referenced to the vertex. Electrode impedance was reduced below 50 kΩ prior to data collection and data were sampled at 500 Hz. An investigator experienced in reading electroencephalograms (SBM, TB, or GV) visually monitored the data to ensure continued signal integrity throughout the experiment. After the experiment, the EEG was bandpass filtered between 0.1 and 50 Hz and re-referenced to an average reference. Epochs and channels with noise or non-physiological artifacts were identified and removed.

### Analysis epochs

**Figure 1:**
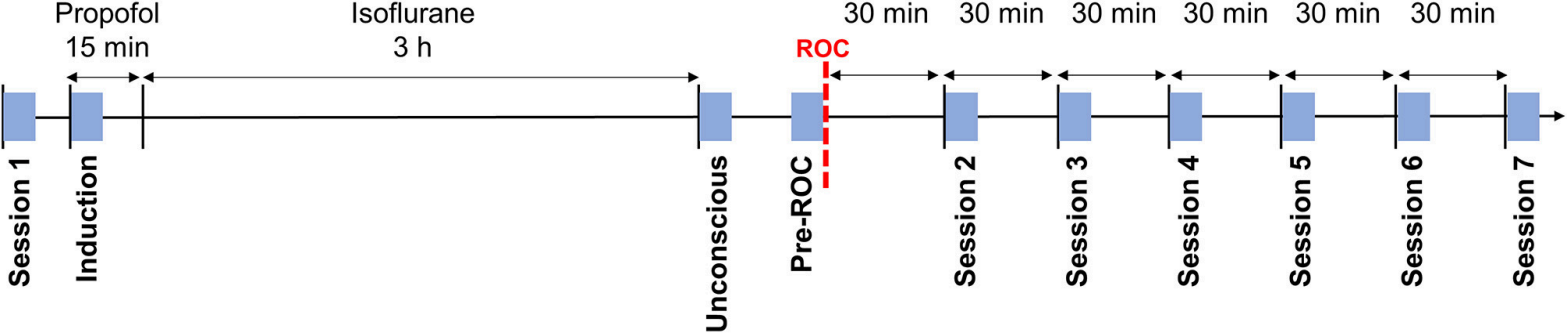
Experimental design and timeline. Blue squares indicate the 5-min analysis epochs distributed throughout the experiment during which participants were asked to rest with their eyes closed. ROC, recovery of consciousness.

During the experiment, participants were in a resting state seven times for 5-min epochs. During these sessions, participants were instructed to remain still with their eyes closed while their EEG was recorded. Participant responsiveness was monitored with the same auditory command used for assessment of the anesthetic state transitions, in order to ensure that they remained awake for the duration of the session. Session 1 occurred prior to the induction of anesthesia. Session 2–7 occurred, respectively, at 30, 60, 90, 120, 150, and 180-min post-ROC. Additionally, three 5-min epochs were extracted during the exposure to anesthesia: (1) “induction”—the first 5-min of exposure to propofol; (2) “unconscious”—the first 5-min after the discontinuation of isoflurane; and (3) “pre-ROC”—the 5-min immediately preceding ROC (Figure 1). Collectively, these define 10 analysis epochs for each participant.

### Electroencephalographic analysis

#### Source estimation

Cortical current source density mapping was calculated using a distributed model consisting of 10,000 current dipoles. Dipole locations, and orientations were constrained to the cortical area of the standard brain model of the Montreal Neurological Institute, which was then warped to the geometry of the sensor net using the Brainstorm software package (Tadel et al., 2011). The EEG forward model was computed using a Symmetric Boundary Element Method from the open-source software OpenMEEG (http://openmeeg.github.io). Cortical current maps were then computed from the EEG time series through a linear inverse estimate (weighted minimum-norm current estimate) using Brainstorm. Finally, the principal-component current activity from within the 68 brain regions defined by the Desikan-Killiany brain atlas were calculated to generate a single time series for each brain region.

#### Spectral analysis

Spectrograms were computed in Chronux (http://chronux.org/) (Mitra and Bokil, 2008) using the multitaper method, with window lengths of *T* = 2 s, step size = 0.1 s, time-bandwidth product *NW* = 2, number of tapers *K* = 3. For each participant, a time series of source-localized activity was generated for four posterior brain regions (precuneus, cuneus, inferior parietal, superior parietal) where many of the brain's network hubs are localized (Moon et al., 2015). For each brain region, the region-based time series from all participants was used to generate group-level spectrograms for each analysis epoch.

#### Functional connectivity

Functional connectivity was assessed using Phase Lag Index (PLI), a measure designed to address the problem of volume conduction by accounting for only nonzero phase lead/lag relationships (Stam et al., 2007). The instantaneous phase of each EEG channel was computed via a Hilbert transform, and the phase difference ΔΦ_t_ between all channel combinations was calculated. PLI was then calculated as follows:

$$PLI_{ij}=\left|\langle sign(\Delta \varphi_{t}\rangle \right|$$

Here, the sign() function yields a value of 1 if ΔΦ_t_ > 0, a value of 0 if ΔΦ_t_ = 0 and a value of −1 if ΔΦ_t_ <0. Thus, PLI quantifies the degree of phase locking of an instantaneous phase relationship. PLI values range between 0 (no locking) and 1 (perfect locking). Each analysis epoch was divided into non-overlapping 10 s windows for which the PLI across all channel combinations was calculated. For each analysis epoch, an average PLI matrix was generated across all 10 s windows and the global functional connectivity was calculated as the average PLI across all channel combinations. PLI was calculated across four frequency bands: (1) delta (1–4 Hz); (2) theta (4–8 Hz); (3) alpha (8–13 Hz); and (4) beta (13–30 Hz).

#### Graph-theoretical analysis

We constructed a brain network using the alpha bandwidth (8–13 Hz) of the EEG, which is the most prominent and frequently studied bandwidth in spectral analysis. The functional network was constructed using the weighted phase lag index (WPLI; Vinck et al., 2011) as follows:

$$WPLI_{ij}=\;\frac{\left|E\{ℑ(C_{ij})\}\right|}{E\{\left|ℑ(C_{ij})\right|\}}=\;\frac{\left|E\left\{\left|ℑ\left(C_{ij}\right)\right|sgn\left(ℑ\left(C_{ij}\right)\right)\right\}\right|}{E\{\left|ℑ\left(C_{ij}\right)\right|\}}$$

where ℑ(*C*_*ij*_) is the imaginary part of cross-spectrum *C*_*ij*_ between two signals *i* and *j*. If the phases of signal *i* always lead or lag those of signal *j*, that is, Pr{*sgn*(ℑ(*C*_*ij*_)) = 1 *or* − 1}, then *WPLI*_*ij*_ = 1. If the phase lead/lag relationship of two signals is random, *WPLI*_*ij*_ = 0.

Next, we constructed a binary adjacency matrix *A*_*ij*_. If the WPLI_*ij*_ value of nodes *i* and *j* was within the top 10% of WPLI-values, *A*_*ij*_ = 1; otherwise, *A*_*ij*_ = 0. From the binary adjacency matrix, we calculated basic network properties, including average path length, clustering coefficient, modularity, and global efficiency. The average path length (L_w_) is the average of the shortest path lengths (L_ij_) between all pairs of nodes in the network (Latora and Marchiori, 2001). The clustering coefficient represents how the nodes of a graph tend to cluster together, with higher values implying networks with highly clustered or regular structures (Watts and Strogatz, 1998). The clustering coefficient (C_w_) for the network was calculated by averaging the clustering coefficients of all individual nodes (C_i_). The modularity of the network represents the sum of connection strengths within modules and was calculated using the Louvain algorithm in the brain connectivity toolbox (Rubinov and Sporns, 2010). High modularity values imply networks with strong within-module connections and weak between-module connections (Newman, 2006). Finally, the global efficiency is the inverse of the average shortest path length over all pairs of nodes.

All network metrics were normalized against randomized networks. Ten random networks were generated by shuffling the empirically-generated network's edges while preserving the degree distributions (Maslov and Sneppen, 2002), which is also known as a null model. The path length and clustering coefficient for each null model were calculated and averaged, yielding L_r_ and C_r_, respectively. Normalized path length (L_w_/L_r_) and clustering coefficient (C_w_/C_r_) were then calculated. The expected connection strength (P_ij_) of the null model was calculated, and subtracted from the modularity, yielding Q—the sum of connection strengths within modules after eliminating null model effects. Normalized global efficiency was calculated from the normalized path length.

### Statistical analysis

Average spectral power, PLI, and the network properties were compared across the 10 analysis epochs. One-way repeated measures ANOVA was applied, with Bonferroni correction of alpha (<0.05) for multiple comparisons of each analysis epoch.

## Results

### Recovery of alpha power in posterior brain regions after emergence from general anesthesia

**Figure 2:**
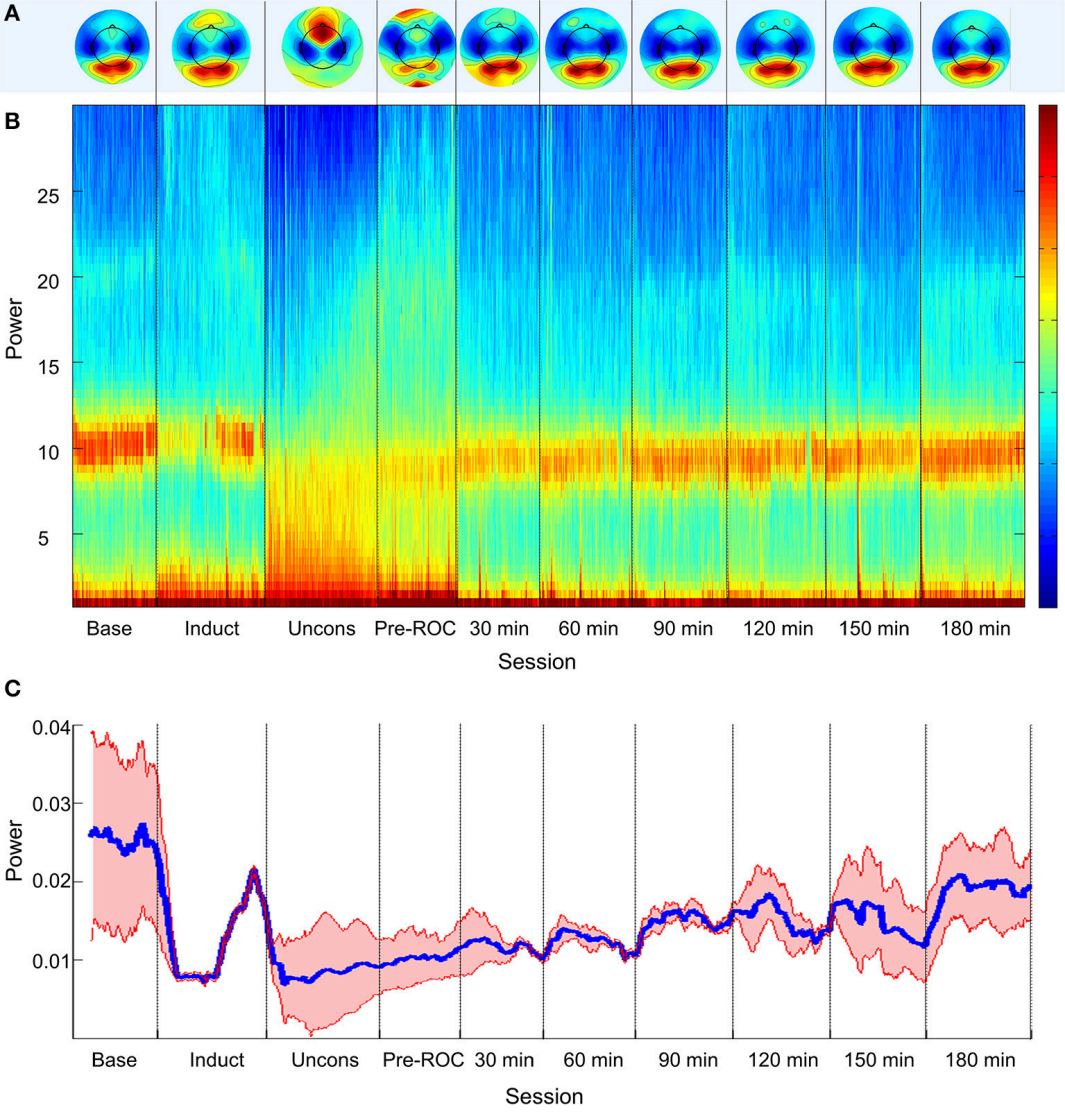
Alpha bandwidth topography and power across ten analysis epochs. **(A)** Topographic mapping of alpha power; **(B)** Source-localized spectrogram of the superior parietal region; and **(C)** Mean alpha power in the superior parietal region (blue) and standard error across all participants (red). Base, Baseline consciousness; Induct, Induction; Uncons, Unconsciousness; ROC, Recovery of consciousness.

Topographic analyses of the spatial distribution of alpha power in all analysis epochs averaged across all eight participants are presented in Figure 2A. Baseline posterior-dominant alpha power shifted to frontal dominance during unconsciousness. This anteriorization of alpha power reversed upon recovery of consciousness and returned to baseline patterns 90-min post-emergence.

The group-level, source-localized spectrogram demonstrated similar trends across all four posterior brain regions. The spectrogram of the bilateral superior parietal region is presented as a representative region in Figure 2B; the superior parietal region was selected because it was associated with the strongest trend of alpha recovery of all posterior sources studied and thus represents the best-case scenario. Alpha power (Figure 2C) decreased significantly upon induction and unconsciousness, exhibiting a gradual but significant increase toward baseline power levels across all analysis epochs post-emergence. Three hours post-emergence, alpha power approached but did not return to baseline levels (*p* < 0.001).

### Phase lag index during states of consciousness or recovery

**Figure 3:**
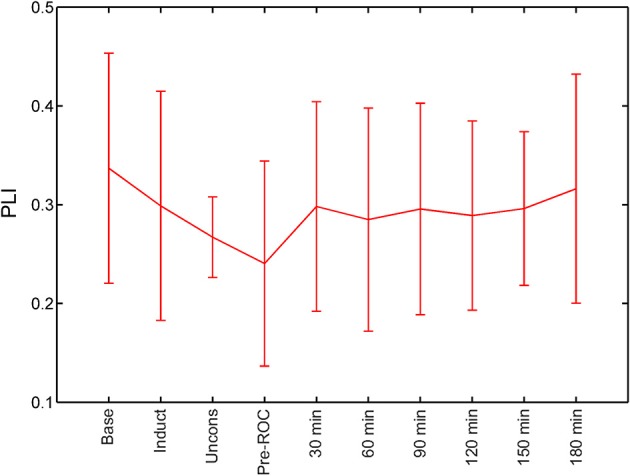
Global functional connectivity calculated using phase lag index (PLI) across all analysis epochs. Error bars represent standard deviation. ROC, recovery of consciousness.

We calculated PLI of global channel combinations in the alpha bandwidth for all analysis epochs (Figure 3). The PLI of each epoch was compared against baseline. PLI did not vary with state of consciousness during induction, unconsciousness, or just before ROC, nor did it vary from baseline at any analysis epoch post-emergence.

### Recovery of functional brain network properties after emergence from general anesthesia

**Figure 4:**
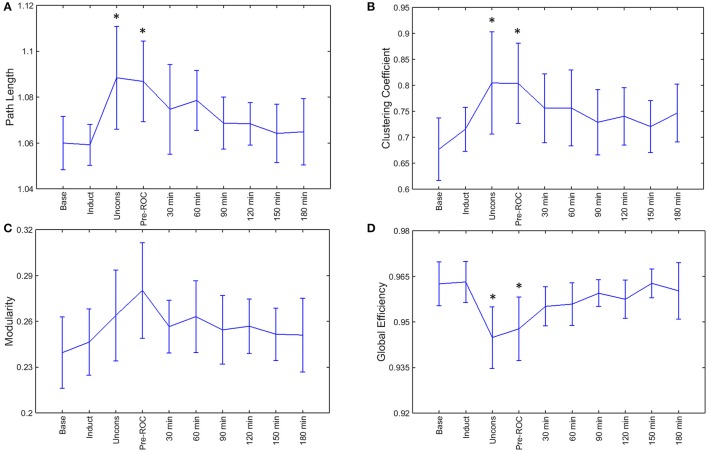
Brain network properties across the experimental period: **(A)** path length; **(B)** clustering coefficient; **(C)** modularity; and **(D)** global efficiency. Error bars represent standard deviation, and ^*^indicates epochs that are significantly different from the baseline (*p* < 0.05). Base, Baseline consciousness; Induct, Induction; Uncons, Unconsciousness; ROC, Recovery of consciousness.

During general anesthesia, brain networks demonstrated a significant increase in path length (*p* = 0.002) and clustering coefficient (*p* = 0.002) as well as a significant decrease in global efficiency (*p* < 0.001) (Figure 4). Changes in network properties persisted until emergence from anesthetic-induced unconsciousness (path length, *p* = 0.005; clustering coefficient, *p* = 0.002; global efficiency, *p* = 0.002). These three network properties did not differ significantly from their baseline values 30-min after recovery of consciousness.

## Discussion

Brain network recovery after a major functional perturbation, such as general anesthesia, is of both clinical and neuroscientific interest. In this study, we found that global functional connectivity patterns using PLI did not distinguish states of consciousness, which is consistent with our prior findings (Lee H. et al., 2013), but global network efficiency dropped during unconsciousness and returned to baseline levels early in the recovery process. Changes in global efficiency were defined by inverse changes in path length, collectively suggesting that—during general anesthesia—information transfer across networks is impeded. Depression of network efficiency and/or surrogates of information transfer have now been identified across a variety of anesthetic drugs (propofol, sevoflurane, ketamine, dexmedetomidine) with diverse molecular and neurophysiological profiles (Boveroux et al., 2010; Lee et al., 2011; Schröter et al., 2012; Lee U. et al., 2013; Jordan et al., 2013; Moon et al., 2015; Palanca et al., 2015; Bonhomme et al., 2016; Ranft et al., 2016; Hashmi et al., 2017). The return of source-localized alpha power during recovery appears to follow a similar trajectory, raising the possibility of a sensor-level biomarker for functional brain network recovery after anesthesia that can be measured in real time and personalized based on the initial posterior alpha power.

Graph theory originated with the early work of Leonhard Euler in the eighteenth century and has been used in the neurosciences to assess brain networks for at least a decade (Ferri et al., 2007, 2008; Spoormaker et al., 2010; Mišić and Sporns, 2016). In 2010 and 2011, graph theory was first applied to the study of anesthetic state transitions by our laboratory (Lee et al., 2010, 2011), demonstrating preservation of some network organizational principles during general anesthesia as well as dissociable network properties of anesthetic induction and recovery. Graph-theoretical analysis has since been applied to neuroimaging and neurophysiologic recordings during general anesthesia in both humans and animals. Of note, the first application of graph theory to neuroimaging during anesthesia (isoflurane in rats; propofol in humans) did not identify significant changes in path length (a determinant of efficiency; Liang et al., 2012; Schröter et al., 2012). However, a more recent study of functional networks reconstructed from 128-channel EEG in the alpha bandwidth—a methodology similar to the current study—found significant increases in path length associated with propofol-induced unconsciousness (Chennu et al., 2016). Furthermore, using fMRI, Monti et al also identified a reversible disruption of path length that returned with the initial recovery of consciousness (Monti et al., 2013). Our work confirms that network efficiency is impaired during general anesthesia, but with an inhaled anesthetic and a clinically-realistic protocol.

The relationship between graph-theoretical analysis and recent efforts to assess information transfer within functional brain networks during general anesthesia is notable. Propofol, sevoflurane, and ketamine have all been found—by both neurophysiologic and neuroimaging studies—to disrupt connectivity patterns and surrogates of information transfer in frontal-parietal networks (Hudetz and Mashour, 2016). It is likely that this line of investigation and network analyses are fundamentally related. First, altered connectivity patterns and network reconfigurations leading to inefficiency would naturally result in impaired information transfer. Even in studies without information-theoretical measures, increased path length and decreased efficiency suggest conditions that might impede information exchange. Second, several lines of evidence support the hypothesis that topology shapes information transfer in large-scale (spatially) and long-scale (temporally) functional brain networks. Past studies of propofol using EEG show that reversal of directional connectivity in frontal-parietal networks parallels a reversal in the topology of hubs (Lee H. et al., 2013). On a more fundamental level, highly-connected network hubs appear to be sinks for information transfer, while peripheral nodes appear to be sources. This has been established in mathematical and simulation studies as well as empirical studies in multiple states and species (Moon et al., 2015, 2017).

Although graph analyses and studies of information transfer are of neuroscientific interest, they are not currently measurable in real time in the routine clinical setting. The finding that alpha power in the superior parietal lobule returns on a more gradual time scale might have clinical relevance. In a large randomized controlled trial that assessed the effectiveness of the bispectral index and end-tidal anesthetic concentration monitoring in preventing intraoperative awareness and improving post-surgical recovery, mean discharge-readiness times in the recovery room were approximately 95-min (Mashour et al., 2012). Thus, the recovery of posterior alpha power in the current study parallels real-world recovery after surgery and anesthesia. Alpha power is known to shift from the posterior region to the frontal area at the point of propofol-induced unconsciousness as well as during surgical anesthesia with sevoflurane (Purdon et al., 2013; Akeju et al., 2014) and (as shown in this study) isoflurane. To date, posterior alpha has typically only been measured upon initial recovery and often only after relatively short exposure. The present findings encourage further study of sensor-level alpha changes in the posterior region that might serve as a marker for recovery from anesthesia. Further, study of the relationship of network-level changes and posterior alpha power is warranted.

Strengths of this study include high-density (128 channel) EEG recording, a clinically-realistic anesthetic regimen, and serial measurement of resting state networks during a prolonged recovery time. Limitations relate to the poorer spatial resolution of EEG compared to other neuroimaging modalities, the restriction of analysis to the alpha bandwidth, the inability to assess subcortical structures, and the coarse-grained analysis of networks that does not capture the fine-scale dynamics (spatially and temporally) of spike-level networks. Furthermore, the ostensible recovery of network efficiency just after emergence could be due to the relatively low number of participants in the study (*n* = 8). Alternatively, it might relate to the inherent variability of emergence from anesthesia that has been identified experimentally (Lee et al., 2011) and clinically (Chander et al., 2014). Finally, ondansetron was administered for the prevention of nausea and vomiting, and it is possible that this affected the EEG-derived network behavior; however, it is unlikely that this would significantly distort the far more dominant effects of 1.3 age-adjusted minimum alveolar concentration of isoflurane administered for 3 h.

In conclusion, a clinically-relevant anesthetic protocol and recovery process demonstrates a significant depression of functional network efficiency that returns in association with the recovery of posterior alpha power over a clinically-relevant timeframe, suggesting a potential biomarker for normal recovery after general anesthesia.

## Author contributions

MA, MK, and GM conceived of and designed the study; SB, GV, and TB acquired EEG data; VT, EJ, PP, GG, and GM served as the clinical anesthesiologists; SB analyzed data; SB, BP, MA, MK, and GM interpreted the data; SB and GM wrote the manuscript; all authors contributed to critical review of the manuscript.

### Conflict of interest statement

The authors declare that the research was conducted in the absence of any commercial or financial relationships that could be construed as a potential conflict of interest.
